# Machine learning shows torsion angle preferences in left-handed and right-handed quadruplex DNAs

**DOI:** 10.1016/j.bpj.2022.08.021

**Published:** 2022-08-22

**Authors:** Kevin Li, Liliya A. Yatsunyk, Stephen Neidle

**Affiliations:** 1 Department of Chemistry and Biochemistry, Swarthmore College, Swarthmore, Pennsylvania; 2 UCL School of Pharmacy, University College London, London, United Kingdom

## Abstract

Left-handed G quadruplexes (LHG4) have been recently discovered as a new class of G quadruplexes. The biological functions of LHG4s are still unknown, but they share a striking resemblance to Z-DNA in their helicity and jagged phosphate backbone. To further understand structural features of the LHG4s that define their left handedness, we have employed human-interpretable machine-learning methods to classify right- and left-handed G4s purely based on torsional angle analysis. Our results reveal the importance of the α, β, δ, and χ angles in left-handed structuring across both Z-DNAs and LHG4s. Our analysis may serve as the first step to understanding the conditions of formation for LHG4s and their potential biological relevance.

## Significance

Our work explores left-handed G quadruplexes, a novel non-canonical DNA structure. Using machine-learning methods, we have demonstrated that certain backbone torsion angles (α, β, δ, and χ) can be used to differentiate between right- and left-handed G quadruplexes as well as other right- (B-DNA) and left-handed (Z-DNA) DNA structures. Our analysis may serve as the first step to understanding the conditions of formation for left-handed G quadruplexes and their potential biological relevance.

## Introduction

**Figure 1:**
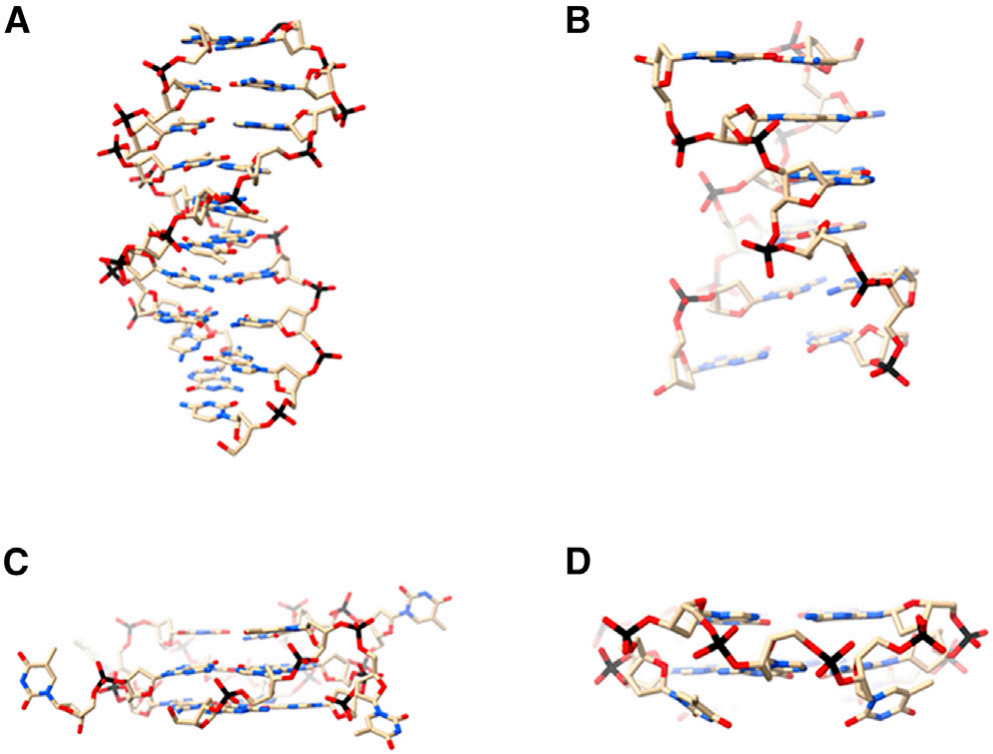
(*A*–*D*) Crystal structures of (*A*) B-DNA (PDB: 1BNA), (*B*) Z-DNA (PDB: 4HIF), (*C*) RHG4 (PDB: 6QJO), and (*D*) LHG4 (PDB: 6QJO). Crystal structure 6QJO contains both right- and left-handed G4s domains. In (*D*), only the LHG4 is shown. To see this figure in color, go online.

Genomic DNA is much more than the encoded blueprint of life. It comprises canonical DNA, which is a right-handed (RH), double-helical (DH) structure, mostly B-DNA, that forms relevant to the majority of cellular DNA functionality (Fig. 1 *A*). DNA, however, can form a variety of non-canonical structures. In regions of high G-C repetition, it may form a DH left-handed (LH) DNA structure, termed Z-DNA (Fig. 1 *B*) (1). Z-DNA is characterized by a jagged phosphate backbone, a feature attributed to the alternating anti-syn dinucleotide steps going from G to C (2). Since its discovery in 1970, the biological relevance of Z-DNA has remained controversial, although in particular the characterization of the ADAR enzyme, an RNA-binding deaminase that binds Z-DNA with high specificity, has suggested a significant biological role for Z-DNA (3). Crystal structures of Z-DNA bound to the Zα domain of ADAR show contacts primarily along the phosphates in the jagged backbone, demonstrating that specificity arises from a Z conformation and not from the DNA sequence (4).

Another non-canonical DNA structural type, termed the G quadruplex (G4), is typically composed of four guanine-rich stretches associated into stacked guanine-tetrads, with RH helicity (Fig. 1 *C*) (5). Numerous RH G4 crystal and NMR structures have been reported (see, for example, refs. (6,7,8,9,10)). Sequences with the potential to form G4s are widely, though not randomly, distributed in many genomes, with their prevalence in the human genome being of special focus (5). Increasing evidence points toward the in vivo existence of G4s, where their presence is implicated in transcription, translation, replication, and telomeric stability (5). Interestingly, the Zα domain from ADAR displays binding affinity and stabilization of a specific conformation of G4—a parallel fold. In this fold, all four G-rich strands are oriented in the same direction (6).

In 2015, the first LH G quadruplex (LHG4; Fig. 1 *D*) was crystallized by the Phan laboratory (7). Its sequence is derived from the guanine-rich RHG4 aptamer AGRO100 (8). To date, eight crystal and two solution NMR structures of LHG4s have been reported, all deriving from the minimal LH motifs of (GTG)_4_ or (GGT)_3_GTG (7,9,10,11,12). These minimal motifs not only fold into LHG4s but are also able to drive LHG4 formation in some related sequences that, alone, would adopt a RHG4 fold (7). The known LHG4 structures consist of two-tetrad G4s, stabilized in the crystal by dimerization with another two-tetrad G4 across a 5′-5′ interface (7). Structurally, LHG4s are characterized by a Z-like jagged backbone oriented in the same direction as its four neighbors (e.g., parallel G4); the thymines that separate each stretch of guanines cap the tops of the tetrads (7). However, unlike Z-DNA, all the nucleotides in LHG4 are oriented in the anti-conformation (7). Genomic searches in the human genome for the LHG4 minimal motifs (GTG)_4_ have returned more than 10,000 hits, which is some two orders of magnitude greater than the expected random occurrence of a 12-bp motif (9). The biological relevance and role of LHG4s is still unknown, as are conditions and driving forces for their formation. Understanding these questions, however, is likely intertwined with understanding LHG4 structures.

We report here studies directed at understanding the structural differences between LHG4s and RHG4s. We have applied decision trees as a human interpretable machine-learning method to analyze torsion angles in both LHG4s and RHG4s. By generating a classifier that discriminates between LHG4s and RHG4s with accuracy of around 90%, we can interpret the produced decision tree to determine the principal torsion angles that distinguish LH and RH. Despite the decision tree never being trained on DH DNA, the methodology was capable of classifying the handedness of Z-DNA and B-DNA with greater than 86% accuracy. After supplementing the training set with Z/B-DNA samples, the accuracy of Z/B-DNA classification increased to 97%. This provides an important check on the ability of the algorithms to reproduce the experimental data and represents the initial part of our analyses.

## Materials and methods

### Data sets

**Figure 2:**
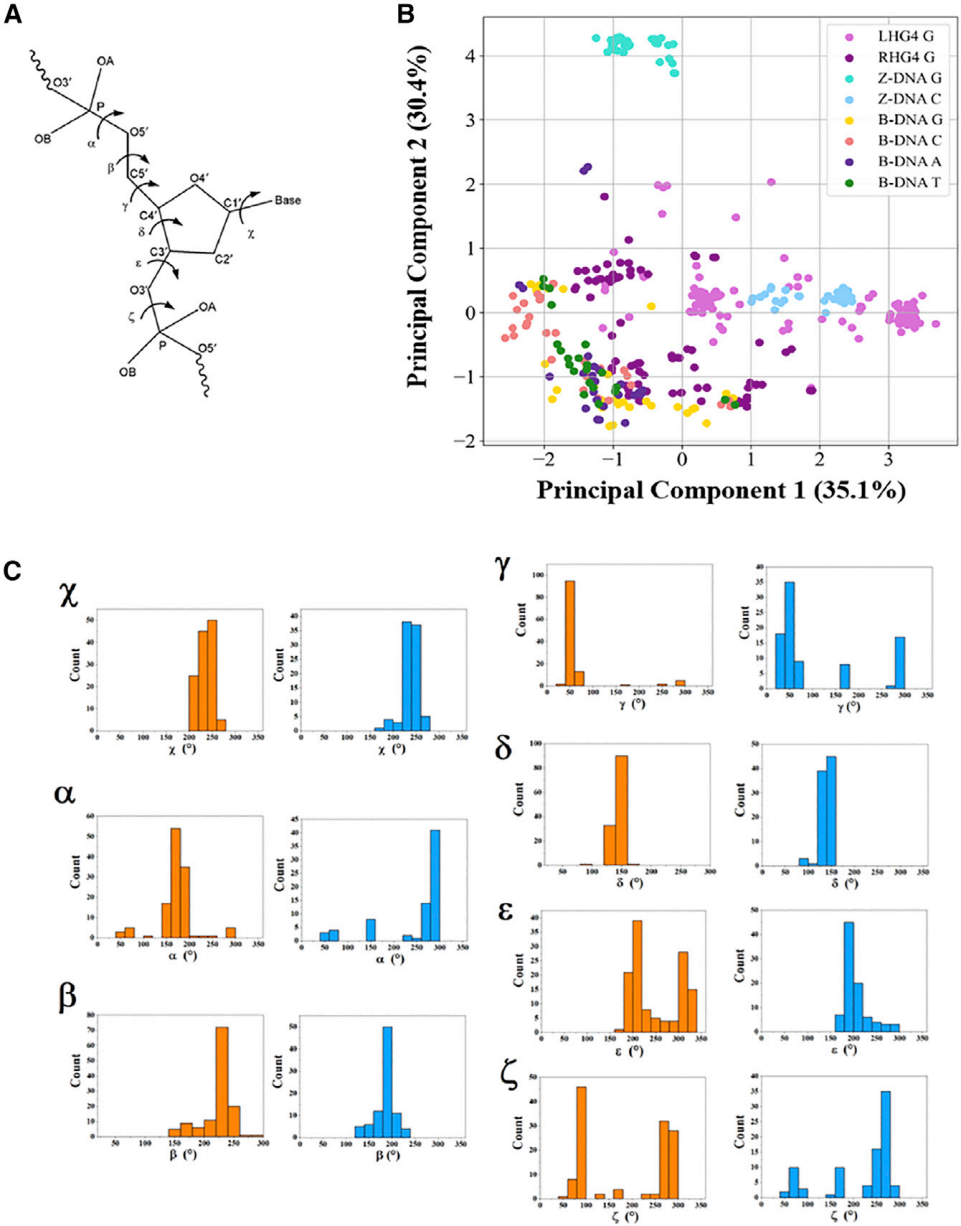
(*A*) Torsion angles considered in this work. (*B*) Two-component PCA of RH and LH DNA structures. Nucleotides are colored based on the structure they come from and their base. (*C*) Torsion angle distribution for guanosines in LHG4 (colored in orange) and RHG4 (colored in blue) across the classification data set. To see this figure in color, go online.

The G4 data set consists of 125 nucleotides taken from seven parallel LHG4 structures and 88 nucleotides taken from six parallel RHG4 structures from the Protein Data Bank (Table 1). Apart from one NMR structure (2N3M), all G4 structures included here had been determined by X-ray crystallography. The DH data set consists of 76 nucleotides taken from eight Z-DNA crystal structures and 175 nucleotides taken from 12 B-DNA crystal structures. These are representative of the highest-resolution structures currently available (an initial study used several early B-DNA crystal structures, with resolutions in the range 1.90–3.00 Å) (Table S1). Each nucleotide sample contains a label of “0” if it originates from an LH structure or “1” from an RH structure. In addition, each nucleotide is characterized by six backbone torsion angles (α, β, γ, δ, ε, ζ) and the base/sugar glycosidic torsional angle (χ) (Fig. 2 *A*). These torsional angles were calculated using the X3DNA web server (36).

**Table 1:** DNA crystal structures included in the data set

PDB ID	Structural type	DNA sequence	Resolution (Å)	References
6FQ2	LHG4	(TG_2_)_4_T_2_(GTG)_4_T_2_	2.31	(11)
7DFY	LHG4	(GTG)_4_	1.69	(9)
4U5M	LHG4	G(TG_2_)_3_TGT_2_(GTG)_4_T	1.50	(7)
6GZ6	LHG4	G_2_T_2_G_2_TGTG_2_T_2_G_2_T (GTG)_4_	2.01	(11)
6QJO^a^	LHG4/RHG4	G(GT)_3_(GGT)_2_(GTG)_4_T_2_	1.80	(12)
7D5D	LHG4	G(GT)_5_G_2_T(GTG)_4_T_2_	1.18	(10)
7D5E	LHG4	(TG_2_)_4_T_2_(GTG)_4_T_2_	1.30	(10)
7KLP	parallel RHG4	A(GGGTTA)_3_GGG	1.35	(13)
6N65	parallel RHG4	AG_3_CGGTGTG_3_AATAG_3_AA	1.60	(14)
3T5E	parallel RHG4	A(GGGTTA)_3_GGG	2.10	(15)
6H5R	parallel RHG4	TA(GGGTTA)_3_GGGT	2.00	(16)
4FXM	parallel RHG4	A(GGGTTA)_3_GGG	1.65	(17)
2N3M^b^	parallel RHG4	(GGT)_3_TGTT(GTG)_3_	–	(18)
3P4J	Z-DNA	CG_3_	0.55	(19)
4OCB	Z-DNA	CG_6_	0.75	(20)
4FS6	Z-DNA	CG_3_	1.30	(21)
4FS5	Z-DNA	CG_3_	1.30	(21)
4HIG	Z-DNA	CG_3_	0.75	(22)
4HIF	Z-DNA	CG_3_	0.85	(22)
7JY2	Z-DNA	CG_3_	1.00	(23)
7ATG	Z-DNA	CG_3_	0.60	(24)
1BNA	B-DNA	(CG)_2_AATT(CG)_2_	1.90	(25)
2BNA	B-DNA	(CG)_2_AATT(CG)_2_	2.70	(26)
3BNA	B-DNA	(CG)_2_AATTC_Br_GCG	3.00	(27)
4BNA	B-DNA	(CG)_2_AATTC_Br_GCG	2.30	(27)
5BNA	B-DNA	(CG)_2_AATT(CG)_2_	2.60	(28)
1D60	B-DNA	CCAACITTGG	2.20	(29)
1SGS	B-DNA	CGCTGGA_3_T_3_CCAGC	1.60	(30)
1DC0	B-DNA	CAT G_3_C_3_ATG	1.30	(31)
1D8G	B-DNA	CCAGTACTGG	0.74	(32)
5DNB	B-DNA	CCAACGTTGG	1.40	(33)
436D	B-DNA	CGCGAA TAF TCGCG	1.10	(34)
4C64	B-DNA	CGCGAATTCGCG	1.32	(35)

a Structure is truncated. 6QJO was split into separate RH and LH components.

b An NMR structure. Only the two tetrad G4s in 2N3M were included.

### Principal-component analysis (PCA)

PCA is a widely used technique for dimensionality reduction, allowing the variance among the higher-dimensionality data set to be represented and visualized in a reduced number of dimensions. This is done by calculating principal components, which are directions in the data set along which the variation is maximized. Data points in the higher dimensionality data set are then projected onto the principal components, producing the dimensionality reduced data set. Plots of the first two principal components are useful in determining interesting clusters of examples that contain similar higher-dimensional characteristics with respect to the principal components.

Principal components were calculated from the torsional angle data set using the PCA class from the scikit-learn Python package, with *n*, the number of components, set to 2 (37). The resultant PCA graph was plotted using the Matplotlib Python package (38).

### Experimental training and validation

Our models were trained on the G4 data set and evaluated using repeated, random holdout validation. Essentially, samples from the G4 data set were randomly split 70/30 into the training and testing sets, such that for the 213 nucleotides in the data set, 149 were randomly placed into the training set, and the remaining 64 were placed in the testing set. The 70/30 training/testing split is commonly used in machine learning (39). Each model then learned the classification by training on the training set and is scored based on its accuracy on the testing set, which contains samples the model has never seen. Accuracy here is defined as the number of samples classified correctly over the total samples in the testing set. This process was repeated 1000 times, each time with different nucleotides randomly split into the training and testing sets to produce a representative average accuracy that is not skewed due to sampling bias. Random holdout validation was performed using the score function from each respective classifier in the scikit-learn package (37).

### ID3 decision trees

The scikit-learn ID3 decision tree is an algorithm that trains by splitting a data set recursively by feature (α, β, γ, δ, ε, ζ, χ) and across a threshold (0 ≤ θ ≤ 360) until similarly classed data points (0 for LH, 1 for RH) are grouped together. The resulting decision tree produced can then be used to classify novel data points. To start, the algorithm finds a node, which is a feature and a threshold, that splits the data set into two resulting sets with the lowest entropy (40). Entropy, in this context, can be understood as a measure of class purity in a data set. The entropy of a data set is defined as $E\left(S\right)=\sum_{i=1}^{c}-p_{i}{\log\;}_{2}p_{i}$, where *c* is the total number of classes (two in this case, LH and RH) and $p_{i}$ is proportion of class i data points over the total data set (40). For data sets of only two classes, entropy values are bounded between 0 and 1. A data set that is purely of one class produces the lowest entropy of 0 (assuming ${\log\;}_{2}0=0$), while a data set that is evenly split between classes produces the highest entropy of 1. After producing two data sets, the algorithm is then recursively called on each resultant data set until the entropy drop between parent and child data sets diminishes below a preset threshold. The ID3 classifier was implemented using the DecisionTreeClassifier class from the scikit-learn Python package with the ccp_alpha parameter set to 0.04 (37). The resultant decision tree was visualized using the plot_tree function from scikit-learn (37).

## Results

### Composition of the data set

The G4 data set consists of 125 nucleotides taken from seven parallel LHG4 structures and 88 nucleotides taken from six parallel RHG4 structures. Our study considers only parallel G4 structures (Table 1) in order to prevent introducing differences in the LH and RH data sets due to G4 topological differences—all the known LHG4 structures have parallel topology. Additionally, we include only guanines forming the tetrads and omit overhang and loop nucleotides since all current LHG4 structures are composed of single thymine loops while the RHG4 structures have longer loop lengths and also contain cytosines, adenines, and guanines in the loop regions. The DH data set consists of 76 nucleotides taken from eight Z-DNA crystal structures and 175 nucleotides from 12 B-DNA crystal structures.

### Nucleotide angle analysis

We analyzed the assembly of backbone and glycosidic torsion angles (Fig. 2 *A*) using two-component PCA (Fig. 2 *B*). The resultant PCA graph captured 65.5% of the variance in the original data set. Overall, nucleotides from Z-DNA and LHG4 each show strong clustering, indicating either strong conformational homogeneity in LH folding nucleotides or low structural variability among the known crystal structures. In LHG4s, the guanosines segregated into two clusters, one populated by the first guanosine in every tract in the 5′-3′ direction and the other populated by the second guanosine (note, all known current LHG4s contain only two guanosines in a tract). Between these values lies the cluster representing cytidines from Z-DNA. The torsional angles of Z-DNA cytidines closely match the guanosines in LHG4, with multiple nucleotides from both clusters of guanosines in LHG4 overlapping with the Z-DNA cytidine cluster. The guanosine Z-DNA cluster, however, is distantly segregated from the rest of the LH clusters. This observation can be explained by the 180° change in the guanosine glycosidic angles, a result of the syn-anti dinucleotide step in Z-DNA.

In contrast to the clustering of the LH nucleotides, RH structures show a large spread of observed torsional angles, with no clear clustering. Despite the wide distribution in RH structures, there is very little overlap between the torsion angles of RH- and LH-derived nucleotides on the PCA plot.

To further investigate the differences between LHG4 and RHG4, we plotted their torsional angle distributions across all the guanosines in the data set (Fig. 2 *C*). Of note, the ε and ζ angles in LHG4 guanosines populate a bimodal distribution unlike their counterparts in RHG4 guanosines. These two angles are principally related to the differences between the first and second LHG4 guanosine in each stretch. Between RHG4 and LHG4 guanosines, their differences in the α angle are most striking. LHG4 guanosines have α angle ranges between 150° and 200°, while α angle ranges for RHG4 guanosines accumulate above 250°.

### Decision trees

**Figure 3:**
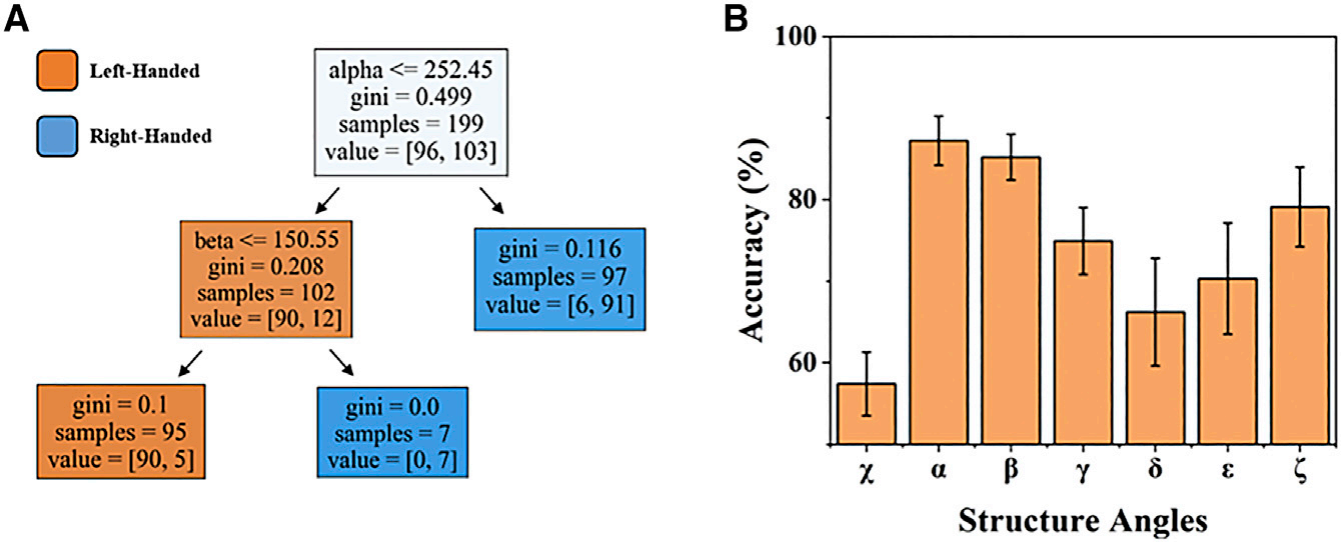
RH versus LH decision tree. (*A*) Decision tree values are an average of the most common decision tree over 10 iterations. Nodes are colored orange or blue based on whether the decision tree would classify the structure as LHG4 or RHG4 if the algorithm halted at that node. The first integer under “value” represents the number of LHG4s in the population, whereas the second integer refers to the number of RHG4s. (*B*) Decision tree accuracy in classifying RHG4 versus LHG4 using single torsion angles. Bar graph shows the average accuracy using each torsional angle over 1000 training-testing iterations. Error bars represent the standard deviation of each accuracy over the iterations. To see this figure in color, go online.

To further understand how DNA torsional angles relate to handedness, we trained a decision tree on the LHG4 + RHG4 data set using the same 70/30 split. This decision tree achieved an accuracy of 89.3% over 1000 iterations. To determine the generalizability of the torsional angle thresholds to right versus left handedness, we then used the same trained model to classify B-DNA versus Z-DNA, which the model had never seen before. Our model achieved an accuracy of 85.6% over the same iterations. These results show a certain degree of transfer learning between the G4 and DH DNA data sets. Over 10 iterations, decision trees trained using only the G4 data set chose to first split on the α angle seven out of 10 times, with α values above 252°, on average, designated as the threshold for RH DNA (Fig 3 *A*). Of these resultant decision trees, all seven unanimously further split on the β angle at 150°. These decision trees perform remarkably well on the DH data set, scoring over 95%. In the remaining three iterations, decision trees chose to split first on the β angle at 215° followed by varying splits in the γ, δ, or ζ angles. These trees score below 65% accuracy on the DH data set, contributing to the 85.6% averaged accuracy.

Motivated by the possibility of transferred learning, we then asked whether adding samples from B- and Z-DNA to the training set would increase the accuracy of the decision tree in classifying LH versus RH DNA (both G4 and DH). Indeed, we observed an increased accuracy in classifying B- versus Z-DNA from 85.6% to 97.7% and LHG4 versus RHG4 from 89.3% to 92.2% over 1000 iterations (Table 2).

**Table 2:** Decision tree accuracy in classifying RH versus LH G4 and DH DNA (B- and Z-DNA)

DH samples added	G4 classifier (%)	DH classifier (%)
0	89.3	85.6
10	90.7	93.1
20	91.5	95.6
30	91.7	96.8
40	92.2	97.4
50	92.2	97.7

As more DH samples (B- or Z-DNA) are added to the training set, the decision trees produced become more likely to split first on the α angle at 252° followed by splitting on the β angle at 150°. After more than 20 DH samples have been randomly mixed into the training set, resultant decision trees unanimously split on the α and β angles at near 252° and 150°, respectively.

In the α and β angle decision tree, the α angle alone already achieves an accuracy of 87.2% in splitting the data set into homogeneous classes. To determine how well the other angles can individually be used to classify LHG4 from RHG4, we then trained decision trees using only values of a single torsional angle at a time (Fig 3 *B*).

Of the seven angles, α and β were most accurate in separating LHG4 and RHG4, scoring 87.2% and 85.2%, respectively. These scores must be compared with the baseline of 58.6% accuracy, which is the percentage of LHG4 in the G4 data set. In other words, arbitrarily guessing LH would still be correct more than half the time.

## Discussion

Patterns of clusters in nucleotide conformations across DNA and RNA structures have been previously identified using Euclidean clustering algorithms (41) and cluster-plot surveys of experimental structures (see, for example, refs. (23,24)). Unlike traditional algorithms where a predetermined set of rules are used to transform an input to the output, machine-learning algorithms are given the input and output of a problem in hopes of formulating the rules that connect them. These algorithms are a powerful tool to parse interesting patterns in a data set. Their application to the nucleic acid field is not a new concept. Particularly in the G4 field, high-throughput studies can generate the massive amounts of data necessary to predict G4 tertiary fold from sequence through neural networks (42). There is considerable current emphasis on deep learning, a branch of machine learning that employs neural networks and multiple layers of data. Deep learning, unfortunately, cannot be yet applied to LHG4 structures due to the lack of data on diverse folds. Only two closely related sequences have been characterized, and the general rules defining LHG4 structures remain to be elucidated. And despite the raw predictive power that deep learning can provide, it is a machine-learning model that sacrifices interpretability for predictive power. Understanding how a neural network comes to its conclusion is typically not at all obvious. Thus, in this study, where understanding how torsion angles correlate with handedness is more important than the prediction itself, we employ ID3 decision trees.

We have coupled our decision tree analysis with more straightforward approaches using PCA plots and histograms. These methods allow a more intuitive understanding of the data set and act as validation for the decision tree output. And while a direct analysis of conformational angles can also reveal trends, the classic approach does not cope well with multi-dimensional data sets, particularly when consideration of multiple angles at once produces a better prediction than single angle analysis. As further instances of LHG4 structures are discovered, a machine-learning approach may be easier to scale to either corroborate existing trends or reveal new ones.

Our decision tree analysis accurately classifies LHG4 versus RHG4 with higher than 92% accuracy by splitting on the α and β angles. Here, guanosines with α angles lower than 252° and β angles higher than 150° are classified as LH, and samples outside these values are classified as RH. These same thresholds, when applied to B- versus Z-DNA, achieve an accuracy up to 97%, demonstrating a strong generalizability between the torsional angles of similarly handed DNA folds.

Analysis of LHG4 versus RHG4 angle distributions corroborates the thresholds made by the decision tree. α values in LHG4s range primarily from 150° to 200°, while those in RHG4s range primarily from 250° to 325°, supporting the threshold of 252° set by the decision tree. The distribution of α angles in LHG4s centers around 180°. This produces a straight-line movement in the backbone progression, creating the signature zig-zag characteristic of the LH fold. Meanwhile, the distribution of α angles in RHG4 peaks at 300°, generating the even and steady curvature of the RH fold. Previous studies have also documented the angle distributions for B- and Z-DNA (41,43): α values of cytidine in Z-DNA closely cluster between 150° and 200°, peaking at 180°, while those of guanosine greatly differ due to the anti-syn step but are lower than 200°. α values in B-DNA match with RHG4, clustering between 240° and 360°. These results are consistent with the PCA plot, which shows overlapping between the RH samples as well as close clustering between cytidines in Z-DNA and the guanosines in LHG4.

Put together, our findings suggest that torsional angle difference between RH and LH DNA structures may be conserved—and, possibly, that the progression of the phosphate backbone may be intrinsically connected to the handedness of the structure. PCA plotting shows overlap between the torsional angles of LHG4 and Z-DNA, which has previously been observed in the analysis of the first LHG4 crystal structure (7). Given that the Zα domain of the ADAR enzyme preferentially binds to the jagged backbone shared between LH structures (Z-DNA and LHG4), it may be interesting to determine whether the Zα domain (4) can also bind LHG4 structures.

Additionally, PCA of the structures covered in this study shows a wide range of torsional angle values for RH structures, contrasting with the tighter clustering produced from LH angles. Although this may be a consequence of the limited diversity of current LHG4 structures, even within the same RHG4 structure, guanosine torsional angles may vary drastically more than within its LH counterpart. The wide spread of the RH torsional values demonstrates a greater degree of flexibility in the backbone, whereas the set of torsional angles where an LH structure can be stabilized appears to be very constrained and rigid, which may explain the low number of available LHG4 scaffolds. An energy landscape of DNA structures based on their torsional angles suggests wide valleys around the torsional values corresponding to RH structures. Meanwhile, the energy landscape around the mean torsional values of LH structures could be imagined as a sharp and narrow dip. For any unfolded DNA polymer rolling around on the energy landscape, it is more likely to take the RH fold as it cascades down the slope of the wide valley than it is to randomly drop into the rigid conformation of the LH fold.

These LH folds are only likely to manifest if conditions arise that make them more stable than their RH counterpart. For DH DNA, it has been shown that under conditions of, for example, negative super coiling, Z-DNA conformations are favored, as the LH helicity acts to relieve some torsional stress (44). Similarly, in sequences that fold into LHG4, it is possible that the LH form is favored because the sequence is unable to arrange the G tetrads in an RH manner. Even so, it is still unknown why LHG4-folding sequences are more adept at folding successive G tetrads into an LH form. These questions may be answered as we further explore the characteristic torsional angles of LH structures using more LHG4 crystal and NMR structures and explore the effect of overhangs on the ability of G4 DNAs to adopt RH or LH folds.

## Author contributions

S.N. came up with the idea and wrote and edited the manuscript; K.L. designed research, performed research, analyzed data, and wrote and edited the manuscript; and L.A.Y. edited the manuscript.

## Supporting material

Document S1. Tables S1 and S2, Figures S1 and S2, and Supporting data

Document S2. Article plus supporting material
