# Supplementary material for: Machine learning shows torsion angle preferences in left-handed and right-handed quadruplex DNAs
Source: Biophys J. 2022 Aug 22;121(24):4874–81. doi: 10.1016/j.bpj.2022.08.021 (PMC9808593; doi:10.1016/j.bpj.2022.08.021)
Supplement: Document S1. Tables S1 and S2, Figures S1 and S2, and Supporting data [file mmc1.pdf]

Biophysical Journal, Volume *121*

Supplemental information

Machine learning shows torsion angle preferences in left-handed and right-handed quadruplex DNAs

Kevin Li, Liliya A. Yatsunyk, and Stephen Neidle

PDB ID	Structural type	Sequence	Resolution (Å)
6FQ2	LHG4	$(TG_2)_4T_2(GTG)_4T_2$	2.31
7DFY	LHG4	$(GTG)_4$	1.69
4U5M	LHG4	$G(TG_2)_3TGT_2(GTG)_4T$	1.50
6GZ6	LHG4	$G_2T_2G_2TGTG_2T_2G_2T$ $(GTG)_4$	2.01
6QJO*	LHG4/RHG4	$G(GT)_3(GGT)_2(GTG)_4T_2$	1.80
7D5D	LHG4	$G(GT)_5G_2T(GTG)_4T_2$	1.18
7D5E	LHG4	$(TG_2)_4T_2(GTG)_4T_2$	1.30
7KLP	Parallel RHG4	$A(GGGTTA)_3GGG$	1.35
6N65	Parallel RHG4	$AG_3CGGTGTG_3AATAG_3AA$	1.60
3T5E	Parallel RHG4	$A(GGGTTA)_3GGG$	2.10
6H5R	Parallel RHG4	$TA(GGGTTA)_3GGGT$	2.00
4FXM	Parallel RHG4	$A(GGGTTA)_3GGG$	1.65
2N3M *	Parallel RHG4	GGT_4	-
3P4J	Z-DNA	CG_3	0.55
4OCB	Z-DNA	CG_6	0.75
4FS6	Z-DNA	CG_3	1.30
4FS5	Z-DNA	CG_3	1.30
4HIG	Z-DNA	CG_3	0.75
4HIF	Z-DNA	CG_3	0.85
1BNA	B-DNA	$(CG)_2AATT(CG)_2$	1.90
2BNA	B-DNA	$(CG)_2AATT(CG)_2$	2.70
3BNA	B-DNA	$(CG)_2AATTC_{Br}GCG$	3.00
4BNA	B-DNA	$(CG)_2AATTC_{Br}GCG$	2.30
5BNA	B-DNA	$(CG)_2AATT(CG)_2$	2.60
1D60	B-DNA	CCAACITTGG	2.20

* *Structures marked with an asterisk are truncated. 6QJO was split into separate RH and LH components. Only the two tetrad G4 in 2N3M was considered.*

Table S1. DNA crystal structures included in the initial dataset

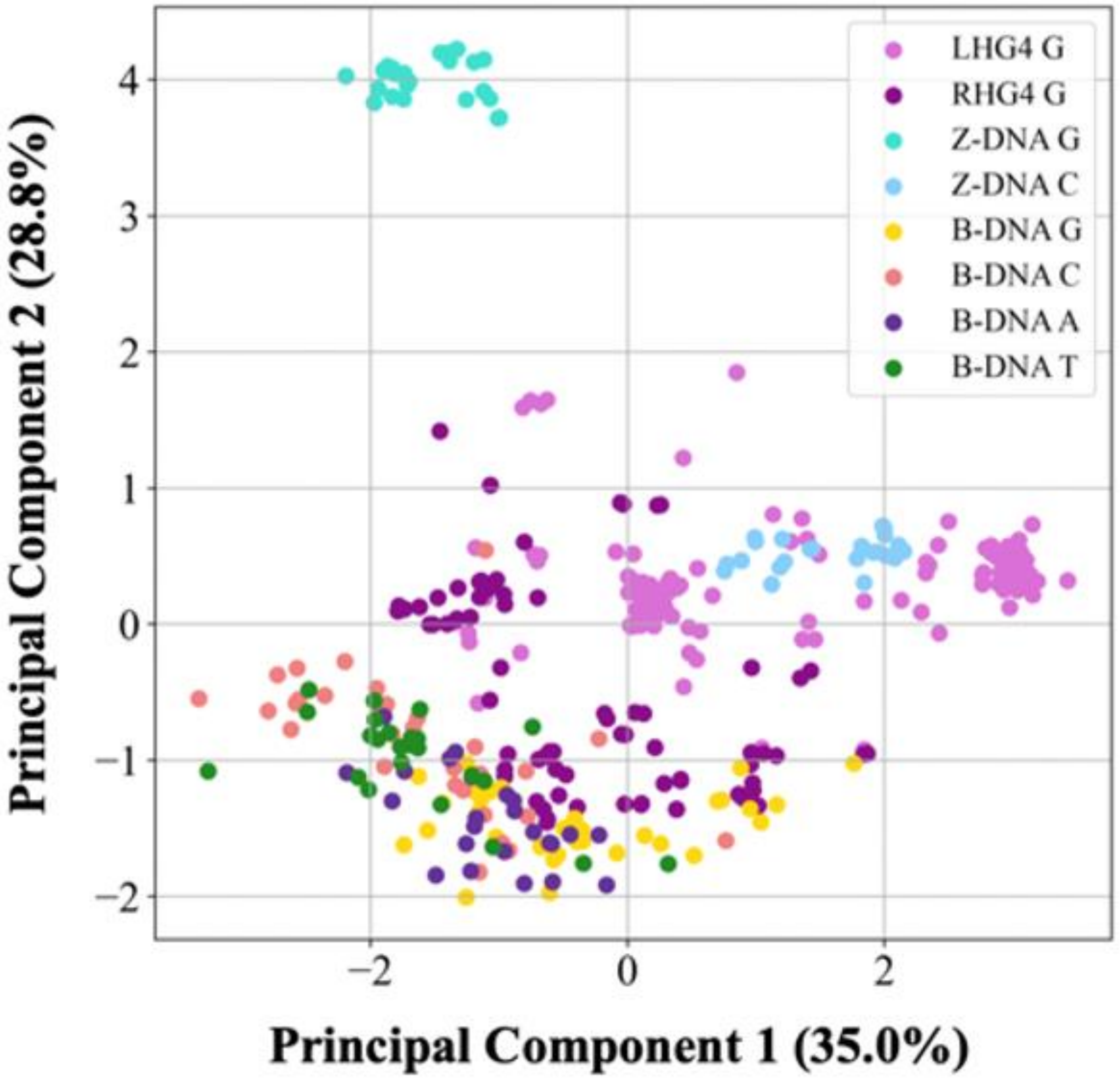

Figure S1: 2-Component PCA of RH and LH DNA structures considered in the initial study. Nucleotides are colored based on the structure they come from and their base. The axis of the PCA plot are two orthogonal vectors in the dimension space of the dataset that capture the most variance in the data.

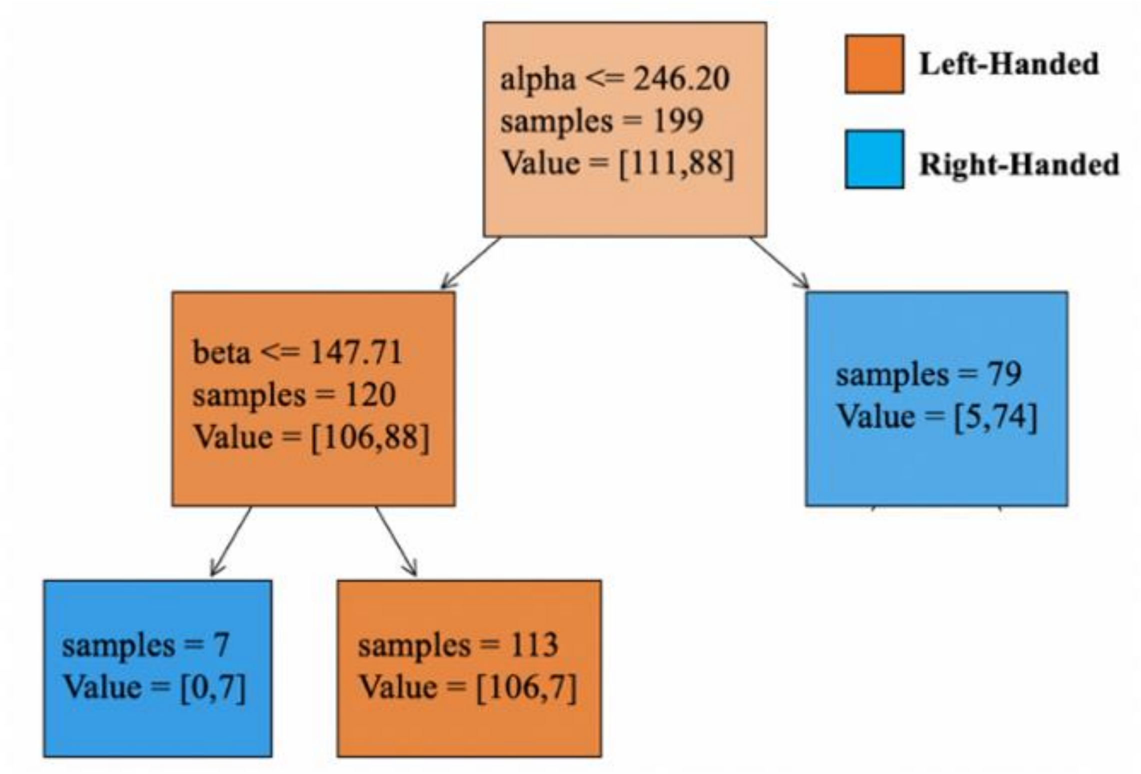

Figure S2: Decision tree after random incorporation of 40 B/Z-DNA samples from the initial dataset. Decision tree values are an average of the most common decision tree over 10 iterations. Nodes are colored orange or blue based on whether the decision tree would classify the structure as LHG4 or RHG4 if the algorithm halted at that node. The first integer under ‘value’ represents the number of LHG4s in the population whereas the second integer refers to the number of RHG4s.

DH Samples Added	G4 Classifier (%)	DH Classifier (%)
0	89.4	79.9
10	92.1	95.0
20	92.2	97.1
30	92.4	97.7
40	92.5	98.0
50	92.6	98.1

Table S2. Decision tree accuracy in classifying right-handed vs left-handed G4 and DH DNA (B- and Z-DNA) using the initial datatset.

Code Supplemental Data

This study will use machine learning techniques to determine interesting structural features of the torsional angles between LH and RH structures.

```
#importing necessary libraries for plotting and machine learning analysis
# import math and numpy first
import warnings
warnings.simplefilter(action='ignore', category=FutureWarning)
import math
import numpy as np
from random import randint
# import scipy for stats package
import scipy
# import some data and classifiers to play with
from sklearn import neighbors
from sklearn import svm
from sklearn import tree
from sklearn.preprocessing import StandardScaler
from sklearn.decomposition import PCA
from sklearn.linear_model import SGDClassifier, LogisticRegression
from matplotlib import pyplot as plt
# import some validation tools
from sklearn.model_selection import train_test_split
from sklearn.model_selection import cross_val_score
from sklearn.model_selection import RepeatedKFold
from sklearn.model_selection import StratifiedKFold, GridSearchCV, validation_curve
from sklearn.metrics import confusion_matrix
#import K-means clustering
from sklearn.cluster import KMeans
import pandas as pd
import random
pd.options.mode.chained_assignment = None
```

Configuring and Loading Dataset

The following structures below were considered in this analysis.

```
LHStructures = ['6FQ2','7DFY','4U5M','6GZ6','6QLH','7D5D','7D5E']
ZStructures = ['3P4J','4OCB','4FS6','4FS5','4FS6','4HIG','4HIF','7ATG','7JY2']
PStructures = ['7KLP','6N65','3T5E','6H5R','4FXM','2N3M']
BStructures = ['1BNA','1D60','2BNA','3BNA','4BNA','5BNA','436D','4C64','5DNB','1D8G',
               '1DC0','1SGS']
```

The torsional angles of each nucleotide in each structure were found using http://web.x3dna.org. The working set is attached below.

```
total = pd.read_excel("TotalDNA.xlsx")
```

Principal Component Analysis

```
#Program allows me to pick specific structures to show the placement of all the
#nucleotides for further analysis.
#This program formats the name of all of the nucleotides so they just contain
#the conformation and what nucleotide it is (i.e LHG4 G). highlighted
#structures will have their nucleotides labeled separately. To highlight:
#if I wanted to highlight the structure 6FQ2 and 1BNA, I would type
#into the console: 6fq2,1bna


def HighlightStructures(total):
    data = total.copy(deep = True)
    Z = ZStructures.copy()
    P = PStructures.copy()
    LH = LHStructures.copy()
    B = BStructures.copy()
    highlightedStructure = []
    print("Highlight specific structures?")
    answer = input()
    if(answer == 'y'):
        print("Type out structures to highlight with comma in between. No Spaces!")
        wantedStruc = input()
        highlightedStructure = wantedStruc.split(',')
        for i in range(len(highlightedStructure)):
            if(highlightedStructure[i] in LH):
                LH.remove(highlightedStructure[i])
            elif(highlightedStructure[i] in Z):
                Z.remove(highlightedStructure[i])
            elif(highlightedStructure[i] in P):
                P.remove(highlightedStructure[i])
            elif(highlightedStructure[i] in B):
                B.remove(highlightedStructure[i])
        for i in range(len(data)):
            if any(structure in data['base'][i] for structure in Z):
                newName = "Z-DNA "+ data['base'][i][6]
                data['base'][i] = newName
            elif any(structure in data['base'][i] for structure in P):
                newName = "RHG4 G"
                data['base'][i] = newName
            elif any(structure in data['base'][i] for structure in LH):
                newName = "LHG4 G"
                data['base'][i] = newName
            elif any(structure in data['base'][i] for structure in B):
```

```python
                newName = "B-DNA "+ data['base'][i][6]
                data['base'][i] = newName
    return data




#Constructing a 2-Component PCA using the dataset and visualizing it with matplotlib.
def RunPCA():
    data = HighlightStructures(total)
    features = ['chi','alpha','beta','gamma','delta','epsilon','zeta']
    print(data)
    x = data.loc[:,features].values
    y = data.loc[:,['base']].values


    x = StandardScaler().fit_transform(x)

    # reduce dimensionality of x to 2 using PCA

    pca = PCA(n_components = 2)

    #calculates principal component axis using transformed data
    principalComponents = pca.fit_transform(x)

    #creating dataframe to contain principal components along with column labels
    principalDf = pd.DataFrame(data = principalComponents, columns =
                               ['principal component 1','principal component 2'])
    finalDf = pd.concat([principalDf, data[['base']]],axis = 1)
    #NucleotidesDf = pd.concat([principalDf,total[['Position']],total['base']],axis = 1)

    #RunPCA.variable = NucleotidesDf


    labels = finalDf.loc[:,"base"].unique()

    #AdjustingPlottingParameters
    font = {'fontname':'Times New Roman'}
    fig = plt.figure(figsize = (8,8))
    ax = fig.add_subplot(1,1,1)
    oneVariance = round(pca.explained_variance_ratio_[0]*100,1)
    twoVariance = round(pca.explained_variance_ratio_[1]*100,1)
    ax.set_xlabel("Principal Component 1 ("+str(oneVariance)+"%)", fontsize = 20)
    ax.set_ylabel("Principal Component 2 ("+str(twoVariance)+"%)", fontsize = 20)
```

```
    ax.tick_params(axis='both', which='major', labelsize=20)
    targets = finalDf.loc[:,"base"].unique()

    colors = ('orchid','darkmagenta','turquoise','lightskyblue','gold',
              'lightcoral','rebeccapurple','forestgreen')
    for target, color in zip(targets,colors):
        indicesToKeep = finalDf['base'] == target
        ax.scatter(finalDf.loc[indicesToKeep,'principal component 1'],
                   finalDf.loc[indicesToKeep, 'principal component 2'],
                   c = color, s = 50)
    ax.legend(targets,prop={'size': 15})
    ax.grid()
    plt.rcParams['font.family'] = 'serif'
    plt.rcParams['font.serif'] = ['Times New Roman'] + plt.rcParams['font.serif']
    plt.savefig('PCA2D.png', dpi = 300)
    #generating saved figure


    plt.show()
```

Decision Tree Classifier

Explanation: I have now taken the dataset and replaced the labels of the nucleotide for 0 and 1. 0 stands for Left-Handed and 1 stands for Right-Handed. We will now use machine learning to see whether we can produce an algorithm that uses the torsional angles to accurate separate left and right handed structures. We can then see which angles the algorithm uses the most to make the distinction to understand what torsional angles are most pivotal in the distinction.

```
#Making G4 only dataset
onlyGQ = total.head(213)
onlyGFeatures = onlyGQ.iloc[:,3:]
onlyGLabels= onlyGQ['LHRH'].head(213)

#Making B-Z DNA dataset, now updated with high resolution B-DNA structures
DHData = pd.read_excel('DoubleDNATEST.xlsx')
DHLabels = pd.DataFrame(DHData.iloc[:,1])
DHFeatures = DHData.iloc[:,3:]

#Making B-Z DNA dataset, with only new high resolution B-DNA structures
#and old Z-DNA structures
newDHData = pd.read_excel('NewDNA.xlsx')
newDHLabels = pd.DataFrame(newDHData.iloc[:,1])
newDHFeatures = newDHData.iloc[:,3:]
```

Adding B/Z-DNA to Decision Tree Training Set

```
def avg(w):
    avg = []
    n = len(w[0])
    for i in range(n):
        curr_avg = 0
        for j in range(len(w)):
            curr_avg += w[j][i]
        curr_avg /= len(w)
        avg.append(curr_avg)
    return avg
```

```
ZBdt = []
nZBdt = []
LHRHdt = []
#z is the number decision tree accuracy iterations
for z in range(1):
    dt = tree.DecisionTreeClassifier(ccp_alpha = 0.04)
    random_seed = random.randint(1,43)
    X_GQtrain, X_GQtest, y_GQtrain, y_GQtest = train_test_split(onlyGFeatures,
                    onlyGLabels, test_size=0.3, random_state=random_seed)
    #uncomment below to use for original+high-resolution B/Z-DNA structures
    X_DHtrain, X_DHtest, y_DHtrain, y_DHtest = train_test_split(
    DHFeatures,DHLabels,
    test_size = 0.3, random_state = random_seed)
    #creates new training test split with only GQs.
    #creates new training test split with only DH.
    for j in range(0):
        #J amount of B/Z-DNA samples are added to the training set
        i =random.randrange(len(X_DHtrain))
        #generate random index in Z-sample set
        DHfeat = X_DHtrain.iloc[i]
        #print(DHfeat)
        X_GQtrain = X_GQtrain.append(DHfeat,ignore_index=True)
        #append features of random Z-DNA to training
        #print(X_GQtrain)
        DHlab = y_DHtrain.iloc[i]
        #print(zlab)
        y_GQtrain = y_GQtrain.append(DHlab)
        #append label of random Z-DNA to sample
```

```
        #print(y_GQtrain)
    dt.fit(X_GQtrain, y_GQtrain)
    LHRHdt.append(dt.score(X_GQtest, y_GQtest))
    ZBdt.append(dt.score(X_DHtest,y_DHtest))
```

```
#print("LHRHacc mean: ", np.mean(LHRHdt))
#print("ZBacc mean: ", np.mean(ZBdt))


#print("LHRHacc min: ", min(LHRHdt))
#print("ZBacc min: ", min(ZBdt))


#fig=plt.figure(figsize=(12,8), dpi= 150, facecolor='w', edgecolor='k')
#tree.plot_tree(dt,feature_names=onlyGFeatures.columns,filled = True,class_names = True)
#plt.rcParams['font.family'] = 'serif'
#plt.rcParams['font.serif'] = ['Times New Roman'] + plt.rcParams['font.serif']
#plt.savefig('DTree.png', dpi = 300)
#plt.show()
```

Decision Tree Single Angle Analysis

```
gqData = pd.read_excel("totalDNA.xlsx")
onlyGQ = gqData.head(213)
onlyGFeatures = onlyGQ.iloc[:,7]
#3 is chi, 4 is alpha, 5 is beta, 6 is gamma, 7 is delta, 8 is epsilon, 9 - zeta
onlyGLabels= onlyGQ['LHRH'].head(213)
DHData = pd.read_excel('DoubleDNATEST.xlsx')
DHLabels = pd.DataFrame(DHData.iloc[:,1])
DHFeatures = DHData.iloc[:,3:]
```
